# Salivary Citrate Deficiency Defines an Endogenous Dentin Remineralization Failure State Through Glandular MT1/2‐Zn^2+^‐ACO2 Dysregulation

**DOI:** 10.1002/advs.78578

**Published:** 2026-10-29

**Authors:** Ran You, Wenhui Wang, Franklin R. Tay, Haotian Dong, Mutong Wei, Ruoyun Wu, Xin Song, Chen Chen, Haifeng Xie

**Affiliations:** ^1^ Department of Operative Dentistry and Endodontics the Affiliated Stomatological Hospital of Nanjing Medical University and School of Stomatology Nanjing Medical University Nanjing Jiangsu People's Republic of China; ^2^ Department of Prosthodontics the Affiliated Stomatological Hospital of Nanjing Medical University and School of Stomatology Nanjing Medical University Nanjing Jiangsu People's Republic of China; ^3^ Department of Endodontics The Dental College of Georgia Augusta University Augusta Georgia USA; ^4^ Department of Stomatology Nanjing First Hospital Nanjing Jiangsu People's Republic of China

**Keywords:** caries susceptibility, citrate metabolism, dentin remineralization, mineral utilization, salivary glands, zinc homeostasis

## Abstract

Rapid caries progression occurs even when salivary flow and calcium phosphate availability are preserved, suggesting a knowledge gap in how available minerals are used for repair. Early‐stage Sjögren's syndrome was examined as a clinical model of this condition. Salivary citrate was markedly lower in the disease cohort and was inversely associated with caries burden, whereas salivary flow and the major mineral ions did not differ from healthy controls. In mineralization systems with matched calcium and phosphate concentrations, the disease‐associated citrate concentration reduced intrafibrillar mineral deposition, disrupted hydroxyapatite organization, and limited structural and mechanical recovery of demineralized dentin. Human salivary gland tissue, an immunization‐induced rat model, and primary salivary gland epithelial cells showed a similar metabolic pattern: increased metallothionein 1/2 (MT1/2), reduced labile Zn^2+^, increased aconitase 2 (ACO2) activity, and citrate depletion. ZnSO_4_ repletion increased intracellular Zn^2+^, reduced ACO2 activity, restored citrate toward control levels, and improved the mineralization activity of epithelial‐cell supernatants. The data indicate that citrate availability influences dentin mineral utilization and associate citrate depletion with glandular MT1/2‐Zn^2+^‐ACO2 dysregulation. These findings support an association between glandular zinc‐citrate dysregulation and impaired remineralization, suggesting that this mechanism may account for impaired endogenous remineralization despite preserved availability of the major mineral ions.

## Graphical abstract

Salivary citrate deficiency in early‐stage Sjögren's syndrome accompanies impaired dentin remineralization despite preserved salivary flow and mineral availability. Matched‐ion experiments reveal disrupted mineral organization and reduced dentin recovery at disease‐associated citrate levels. Glandular MT1/2–Zn^2^^+^–ACO2 dysregulation accompanies citrate depletion, while zinc repletion restores citrate and improves mineralization, highlighting citrate availability as a key factor in mineral utilization.

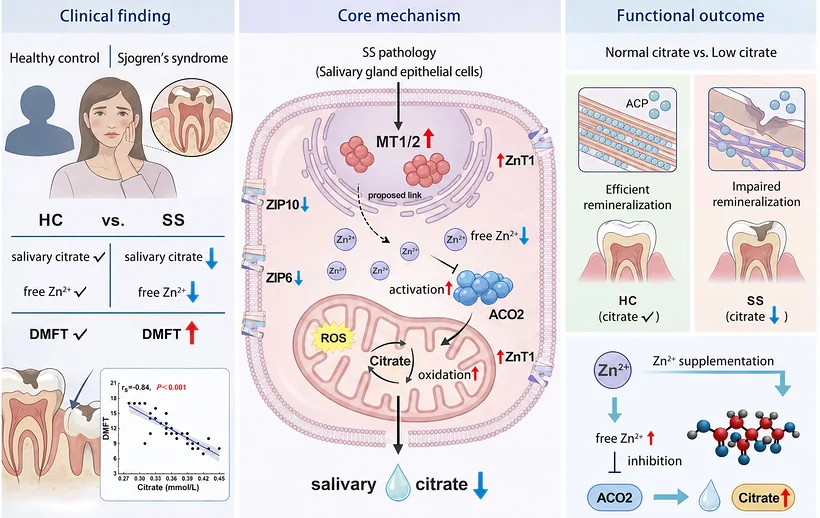

## 1 Introduction

Dental caries develops when mineral loss exceeds repair over time [1, 2, 3]. Biofilm‐derived acids initiate demineralization, while subsequent lesion behavior also depends on salivary and dentin‐matrix conditions that permit mineral replacement [4, 5]. Preventive and restorative approaches consequently emphasize calcium, phosphate, fluoride, and control of microbial acid production [6, 7]. Nevertheless, rapid lesion progression has also been observed when salivary flow and major mineral ion availability are relatively preserved [8, 9]. Such cases raise the possibility that the defect lies in mineral utilization and not simply in mineral supply.

Sjögren's syndrome (SS) offers a useful setting for examining this possibility [10, 11, 12, 13]. Xerostomia is common in advanced SS, although substantial caries can develop before severe hyposalivation is evident [14, 15]. This earlier stage allows salivary composition to be considered with less confounding from a major loss of salivary volume [16, 17, 18]. Early‐stage SS was therefore selected as a translational phenotype for examining whether available calcium and phosphate are effectively converted into organized dentin mineral [19, 20, 21]. The study focused on whether a composition‐dependent defect in mineral use can occur while bulk mineral supply is preserved.

Citrate is a candidate regulator of this process. It is present in saliva and mineralized tissues and interacts with collagen and calcium phosphate precursors [22, 23, 24]. Citrate can alter collagen surface charge, stabilize amorphous calcium phosphate (ACP) [25], influence hydroxyapatite nucleation, and facilitate ordered intrafibrillar mineral deposition [26, 27]. A lower salivary citrate concentration could therefore disturb precursor handling and mineral organization without changing the total calcium or phosphate pool. Its clinical relevance and the metabolic basis for salivary citrate depletion have not been defined.

Salivary citrate may also reflect mitochondrial metabolism in glandular epithelial cells. Aconitase 2 (ACO2) converts citrate to isocitrate [27, 28], and intracellular Zn^2+^ can restrain aconitase activity in zinc‐accumulating cellular contexts [29, 30, 31]. Metallothioneins (MTs), particularly MT1 and MT2, buffer intracellular Zn^2+^ and interact with zinc‐transport systems [32, 33, 34]. Dysregulation of metallothioneins and zinc transport could reduce the labile Zn^2+^ pool, increase citrate oxidation, and lower the amount of citrate available for secretion. A direct connection between this intracellular state and extracellular dentin remineralization has not yet been established. Here, “citrate deficiency” denotes lower salivary citrate relative to controls, whereas “citrate depletion” denotes lower tissue or cellular citrate. “Mineral utilization defect” describes impaired mineral organization under matched ion conditions; “remineralization failure state” is descriptive, not a proven causal endotype.

The study tested whether disease‐associated citrate deficiency can reproduce features of a remineralization defect under mineral‐replete conditions. Clinical association analyses were combined with collagen and dentin mineralization models, human salivary gland tissue analyses, an immunization‐induced rat model, and primary salivary gland epithelial‐cell experiments. Two questions were addressed: whether citrate concentration directly affects dentin mineral organization, and whether glandular MT1/2‐Zn^2+^‐ACO2 dysregulation is associated with citrate depletion. This design links salivary gland metabolism with the extracellular mineralization process.

## 2 Results

### 2.1 Lower Salivary Citrate Correlates with Greater Caries Burden Despite Preserved Salivary Flow and Mineral Ions

The clinical cohort comprised 35 women with early‐stage SS and 35 age‐ and sex‐matched healthy controls (HCs). Unstimulated and stimulated salivary flow rates were comparable between groups, as were salivary Ca^2+^, Mg^2+^, PO_4_^3−^, and HCO_3_^−^ concentrations. Salivary citrate and Zn^2+^, in contrast, were markedly lower in the SS cohort (both *p* < 0.001; Table 1). The difference was therefore selective for citrate and Zn2+ and did not reflect measurable depletion of the major ions required for hydroxyapatite formation.

**TABLE 1:** Salivary flow and composition in the clinical cohorts.

Parameter	SS group (*n* = 35)	HC group (*n* = 35)	*p* value
Unstimulated salivary flow rate (mL min^−1^)	0.31 ± 0.10	0.35 ± 0.07	0.200
Stimulated salivary flow rate (mL min^−1^)	1.26 ± 0.20	1.33 ± 0.15	0.226
Ca^2+^ (mmol L^−1^)	2.52 ± 0.11	2.54 ± 0.14	0.645
Mg^2+^ (mmol L^−1^)	0.31 ± 0.06	0.32 ± 0.09	0.555
PO_4_^3−^ (mmol L^−1^)	14.88 ± 0.96	14.96 ± 0.92	0.774
HCO_3_^−^ (mmol L^−1^)	24.56 ± 2.18	24.92 ± 1.96	0.504
Zn^2+^ (mg L^−1^)	0.56 ± 0.10	1.46 ± 0.17	< 0.0001
Citrate (mmol L^−1^)	0.36 ± 0.03	1.78 ± 0.04	< 0.0001

Data are mean ± standard deviation.

Abbreviations: SS, Sjögren's syndrome; HC, healthy control.

The clinical sampling and analysis workflow is outlined in Figure 1A. In the SS cohort, Spearman analysis showed that lower salivary citrate levels were associated with higher DMFT scores and with greater numbers of decayed (DT), missing (MT), and filled (FT) teeth. No significant citrate‐caries association was detected in HCs (Figure 1B–I). In exploratory XGBoost and SHAP analyses, citrate ranked as the strongest predictor of DMFT among the measured salivary variables in the SS cohort (Figure 1J–L). In contrast, salivary flow variables contributed most strongly in HCs (Figure 1M–O). The modest cohort size limits interpretation of these machine‐learning analyses to hypothesis generation. Overall, the clinical observations describe a low citrate phenotype associated with greater caries burden despite preserved salivary flow and major mineral‐ion concentrations.

**FIGURE 1:**
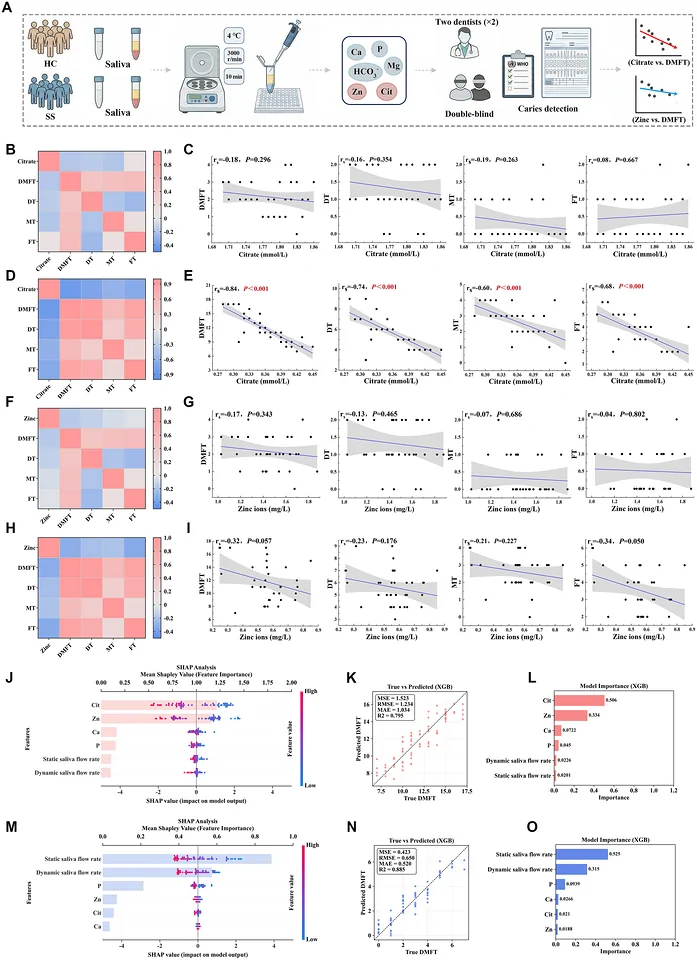
Salivary citrate is associated with caries burden in the disease cohort and dominates exploratory machine‐learning prediction. (A) Clinical sampling and analysis workflow. (B, C) Correlation matrix and Spearman analyses of salivary citrate versus DMFT, DT, MT, and FT in HCs. (D, E) Corresponding citrate analyses in the SS cohort. (F, G) Correlation matrix and Spearman analyses of salivary Zn^2+^ versus caries indices in HCs. (H, I) Corresponding Zn^2+^ analyses in the SS cohort. (J) SHAP summary of salivary predictors in the SS cohort. (K) Observed‐versus‐predicted DMFT for the SS XGBoost model. (L) XGBoost feature importance in the SS cohort. (M–O) Corresponding SHAP, observed‐versus‐predicted, and feature‐importance analyses in HCs. Correlation coefficients and *p* values are shown in the panels; *n* = 35 per cohort. DMFT, decayed‐missing‐filled teeth; DT, decayed teeth; MT, missing teeth; FT, filled teeth; HC, healthy control; SS, Sjögren's syndrome; SHAP, Shapley additive explanations.

### 2.2 Citrate Alters Intrafibrillar Collagen and Dentin Mineralization under Matched Mineral‐Ion Conditions

The functional consequence of the clinical citrate difference was examined in collagen fibrils and demineralized dentin slices under identical calcium phosphate conditions. The primary citrate concentrations matched the mean values measured in HC saliva (1.78 mmol L^−1^) and SS saliva (0.36 mmol L^−1^). Intermediate concentrations were tested in parallel and are reported in Figures S1–S6.

Polyaspartic acid (PAsp)‐ACP particles formed under both citrate conditions, although their association with collagen differed substantially (Figure 2). The low citrate condition produced sparse, discontinuous deposits and limited filling of intrafibrillar and interfibrillar spaces. At the HC‐associated citrate concentration, transmission electron microscopy and elemental mapping showed denser calcium phosphate deposition. Atomic force microscopy also showed greater mineral accumulation and local mechanical recovery. 3D stochastic optical reconstruction microscopy revealed more extensive intrafibrillar mineralization. The concentration of citrate therefore affected both the amount of mineral deposited and its distribution within collagen.

**FIGURE 2:**
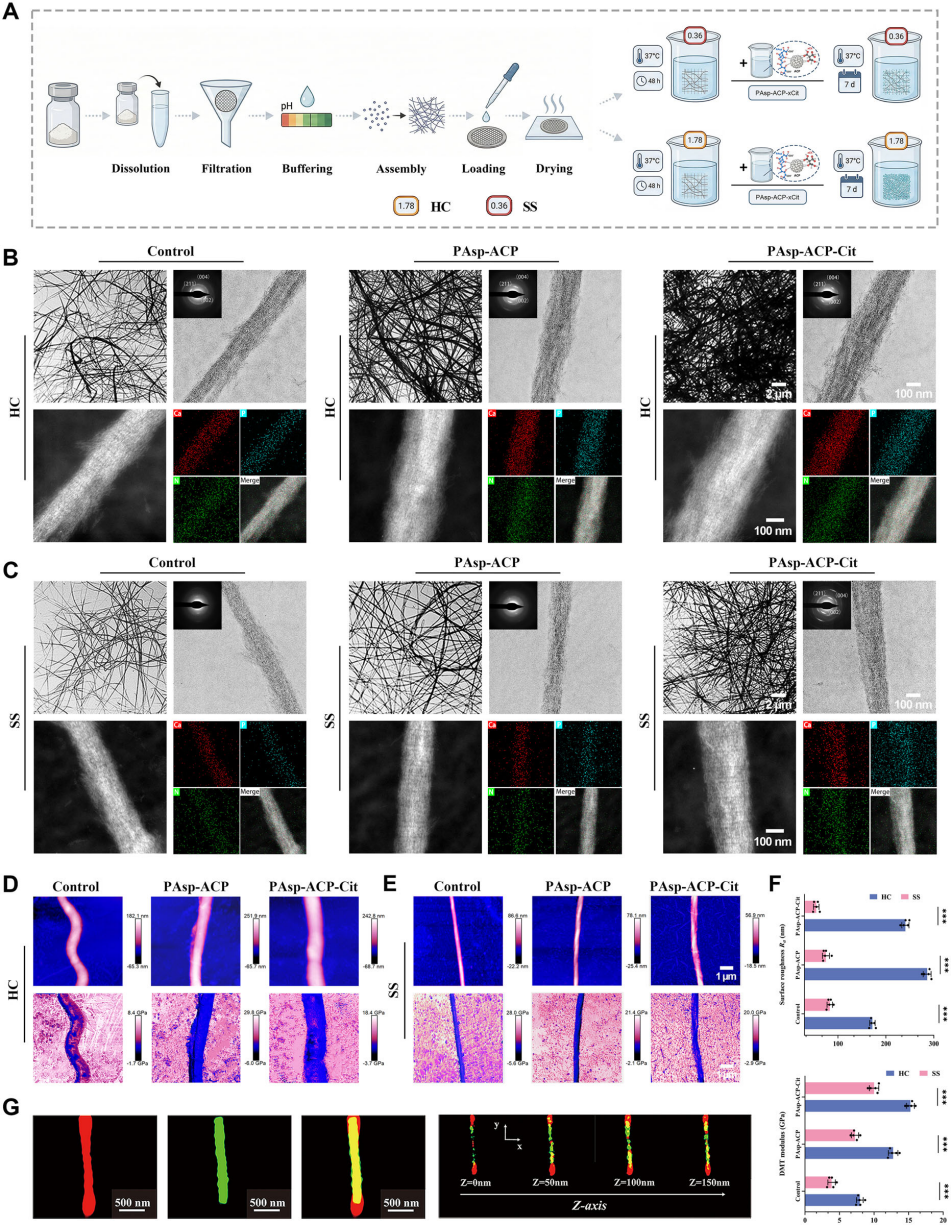
Citrate promotes organized mineral deposition within type I collagen fibrils. (A) Experimental workflow. (B, C) TEM, selected‐area electron diffraction, and elemental mapping of collagen mineralized under HC‐associated (1.78 mmol L^−1^) or SS‐associated (0.36 mmol L^−1^) citrate conditions (0.36 and 1.78 mmol L^−1^ correspond to the mean salivary citrate concentrations measured in patients with Sjögren's syndrome and healthy controls, respectively). (D,E) AFM topography and modulus maps. (F) Quantification of roughness and Derjaguin‐Muller‐Toporov modulus. (G) 3D stochastic optical reconstruction microscopy showing intrafibrillar mineral distribution. Control, mineralization blank; PAsp‐ACP, polyaspartic‐acid‐stabilized amorphous calcium phosphate without added citrate; PAsp‐ACP‐Cit, corresponding citrate‐containing system. Data are mean ± standard deviation from three independent experiments, with at least three distinct collagen fiber samples selected per experiment. Statistical tests and exact *p* values are indicated in the panels.

A similar concentration‐dependent response was observed in demineralized dentin (Figure 3). Semi‐quantitative X‐ray diffraction analysis showed a relative crystallinity of 134.7 ± 3.7% and an average hydroxyapatite grain size of 30.2 ± 0.8 nm at the HC‐associated citrate concentration. Corresponding values in the low citrate condition were 71.0 ± 2.4% and 16.4 ± 0.9 nm. Fourier‐transform infrared spectroscopy showed greater recovery of phosphate‐associated signals with preservation of the collagen amide‐I profile. Scanning electron microscopy and atomic force microscopy further showed denser surface mineral, greater dentinal‐tubule occlusion, lower roughness, and a higher local modulus at the higher citrate concentration. Thus, lowering citrate alone impaired the use of mineral precursors despite matched mineral‐ion concentrations.

**FIGURE 3:**
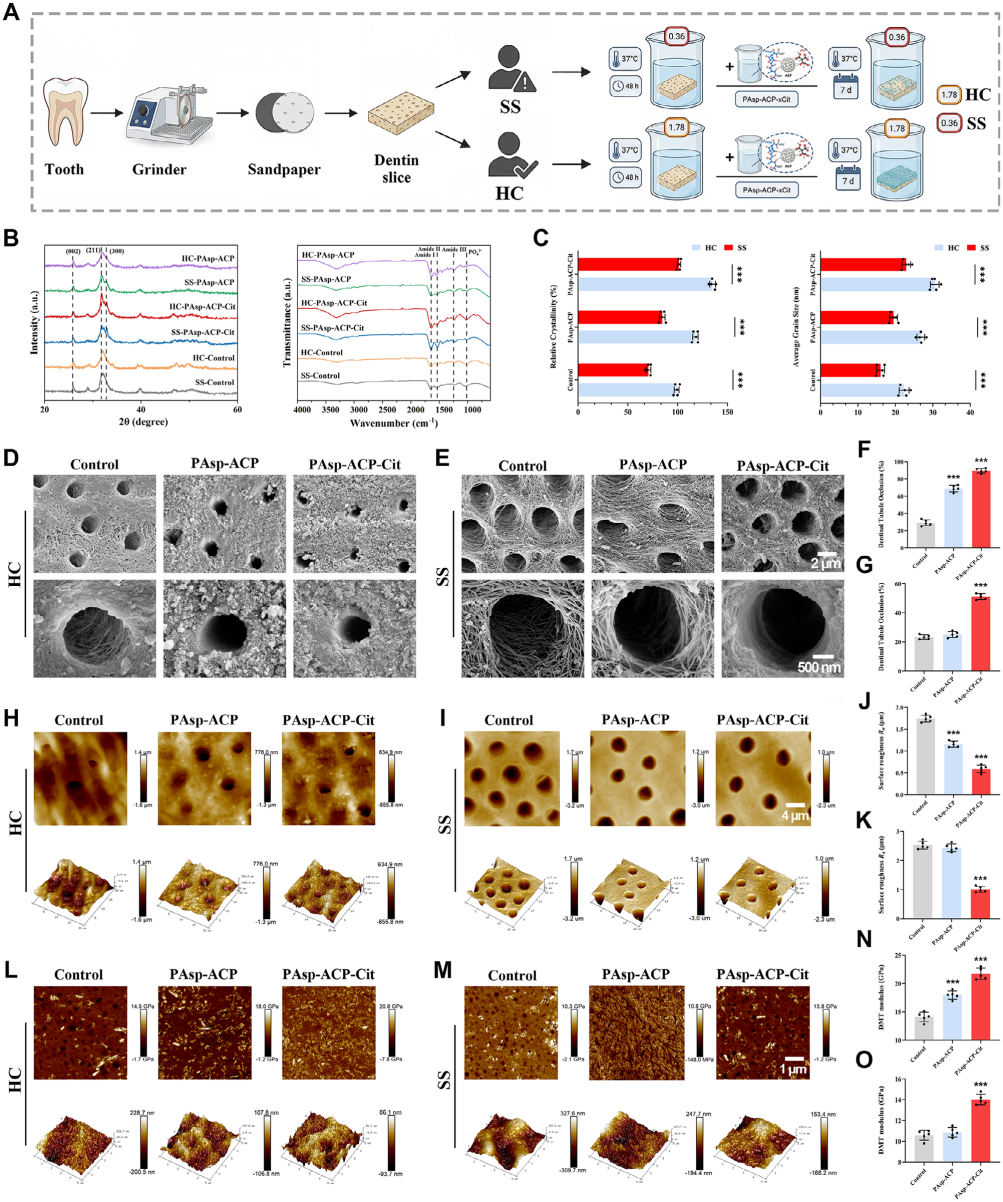
Citrate concentration determines the structural and mechanical recovery of demineralized dentin. (A) Dentin‐disk preparation and mineralization workflow. (B) X‐ray diffraction patterns (left) and Fourier‐transform infrared spectra (right) under HC‐associated and SS‐associated citrate conditions (0.36 and 1.78 mmol L^−1^ correspond to the mean salivary citrate concentrations measured in patients with Sjögren's syndrome and healthy controls, respectively). (C) Quantitative analysis of relative crystallinity and average grain size based on XRD data. (D, E) SEM of dentin surfaces. (F,G) Quantitative analysis of dentinal tubule occlusion calculated from SEM images. (H,I) AFM surface topography. (J,K) Surface‐roughness quantification. (L,M) Derjaguin‐Muller‐Toporov modulus maps. (N,O) Modulus quantification. Intermediate citrate conditions are reported in Figures S1–S6. Data are mean ± standard deviation from three independent experiments, with at least three dentin slices from different extracted teeth selected per experiment. One‐way analysis of variance with Tukey's multiple‐comparison test was used where indicated.

### 2.3 Salivary‐Gland MT1/2‐Zn^2+^‐ACO2 Changes Accompany Citrate Depletion

The human salivary‐gland transcriptomic dataset GSE154926 showed coordinated changes in metallothionein and zinc‐transport genes in SS tissue. MT1G, MT1F, MT1E, MT1L, MT1M, and MT2A were increased, while several ZIP and ZnT family members were altered (Figure 4A,C). Minor salivary gland specimens from the SS cohort also showed higher epithelial MT2 and ACO2 signals by immunohistochemistry (Figure 4D,E) and immunofluorescence (Figure 4H).

**FIGURE 4:**
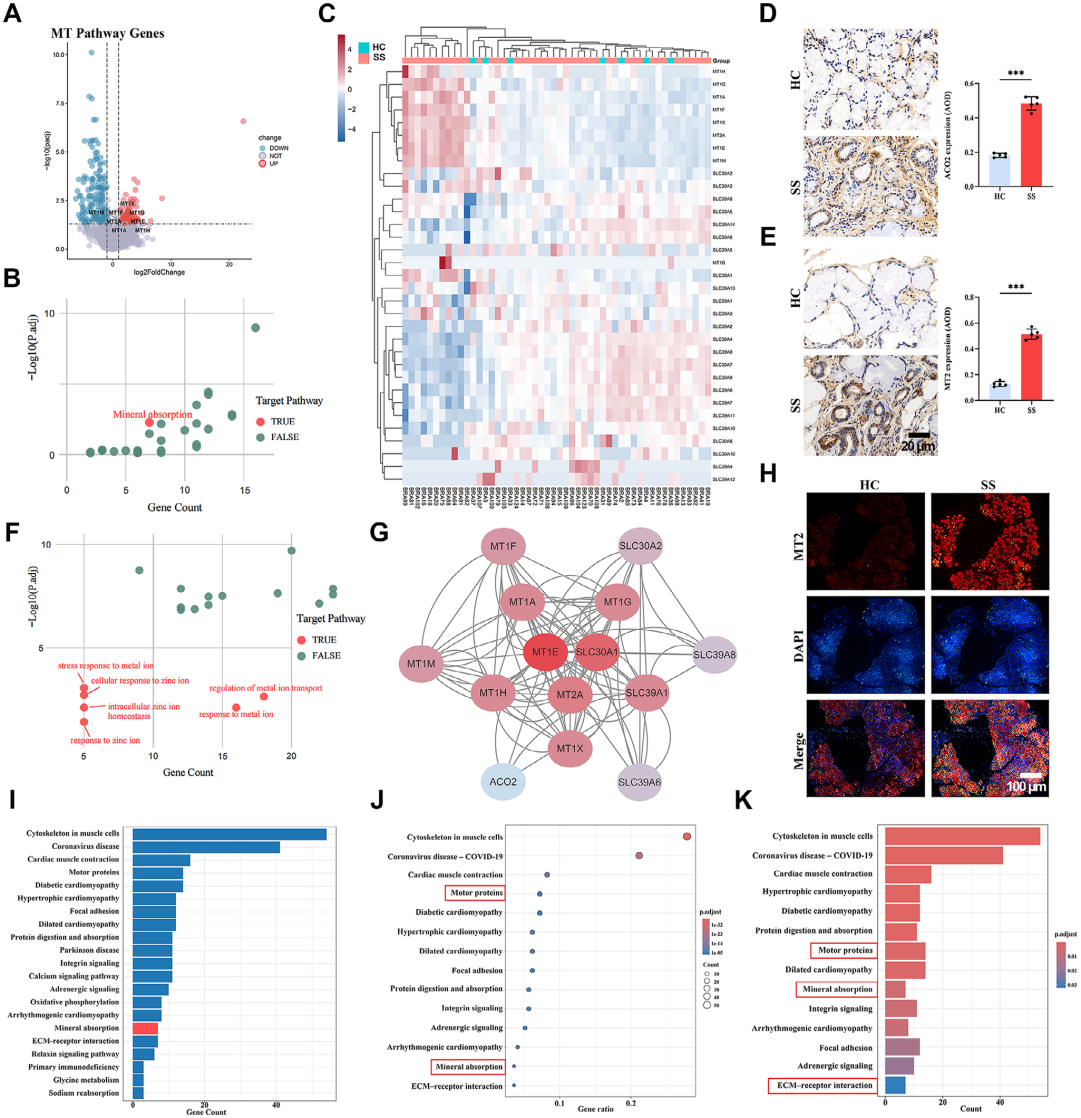
Human salivary gland analyses identify an MT‐Zn^2+^‐ACO2 metabolic module. (A) Volcano plot highlighting metallothionein‐family genes in GSE154926. (B) Kyoto Encyclopedia of Genes and Genomes enrichment highlighting mineral absorption. (C) Heatmap of zinc‐transport and related genes. (D, E) Representative immunohistochemistry and quantification of ACO2 and MT2 in minor salivary glands. (F) Gene Ontology enrichment of metal‐ion‐related biological processes. (G) STRING interaction network containing MT‐family proteins, zinc transporters, and ACO2. (H) Representative MT2 immunofluorescence. (I–K) Additional pathway‐enrichment visualizations. HC, healthy control; SS, Sjögren's syndrome; ACO2, aconitase 2; MT2, metallothionein 2. Tissue samples for immunohistochemistry and immunofluorescence were paraffin sections of human minor salivary glands, with at least five donor samples and over three valid observation regions per sample.

Functional enrichment included metal ion homeostasis, mineral absorption, mitochondrial metabolism, and related pathways (Figure 4B,F,I–K). Search Tool for the Retrieval of Interacting Genes/Proteins (STRING) analysis placed MT‐family proteins, zinc transporters, and ACO2 within the same interaction network (Figure 4G). These results describe an MT1/2‐Zn^2+^‐ACO2 module associated with glandular citrate depletion. However, these analyses alone cannot determine the sequence of events within that module.

### 2.4 The Rat Model Exhibits a Glandular Zinc‐Citrate Metabolic Phenotype

The immunization protocol and tissue collection and analysis workflow are outlined in Figure 5A,B. An immunization‐induced SS rat model was then examined for the same glandular metabolic features in vivo (*n* = 15 per group). Model rats had higher water intake, lower pilocarpine‐stimulated salivary secretion, and lower salivary citrate and Zn^2+^. Salivary calcium and phosphorus did not differ from controls (Table 2). Histology showed inflammatory infiltration and acinar injury in the parotid and submandibular glands (Figure S7). The zinc‐citrate abnormality was reproduced in the model, although the accompanying reduction in salivary secretion differed from the preserved‐flow phenotype of the human cohort.

**FIGURE 5:**
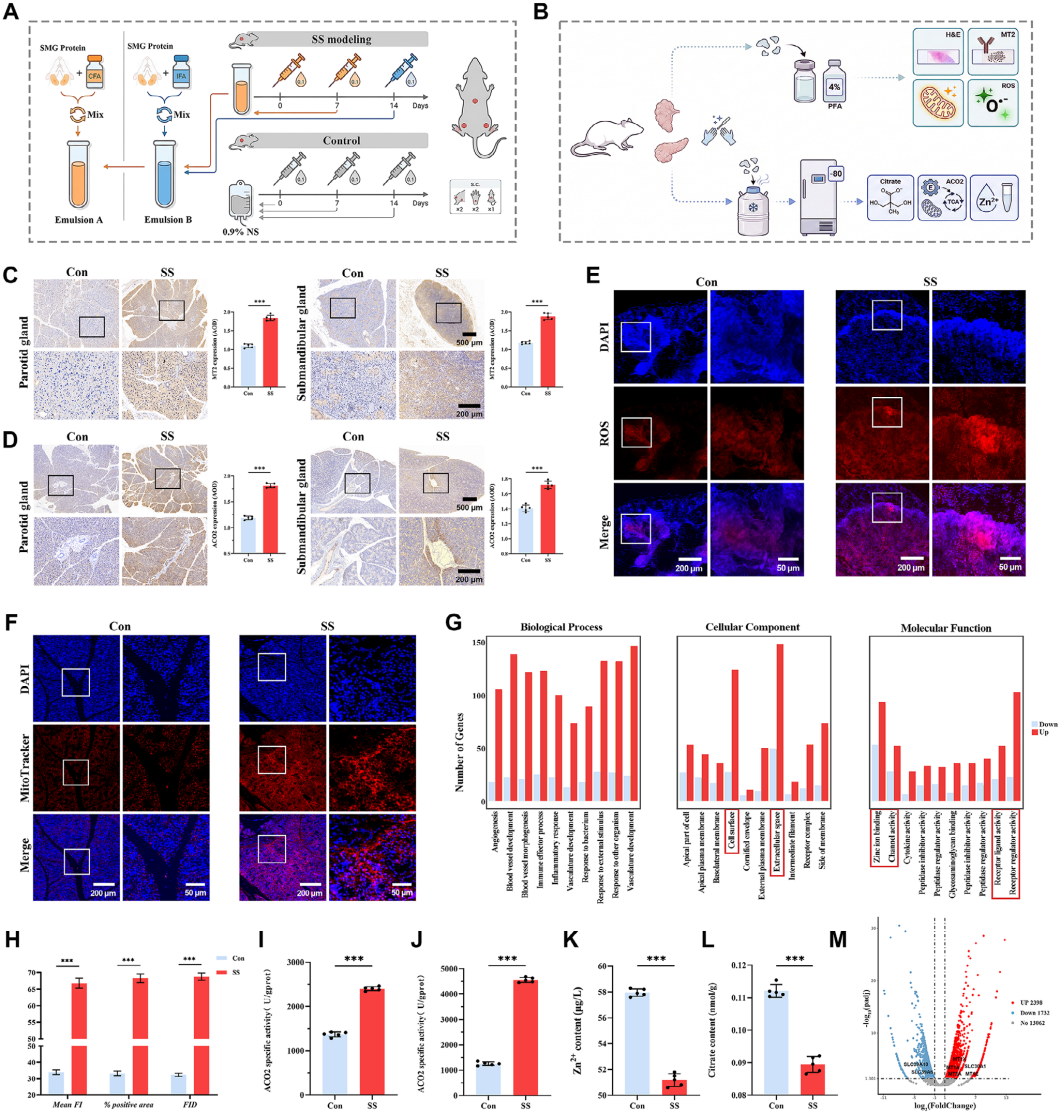
The immunization‐induced rat model recapitulates salivary gland zinc‐citrate metabolic dysregulation. (A) Immunization protocol. (B) Tissue collection and analysis workflow. (C,D) Representative immunohistochemistry and quantification of MT2 and ACO2 in parotid and submandibular glands. (E) Reactive oxygen species imaging. (F) MitoTracker imaging. (G) Gene Ontology enrichment of rat salivary‐gland transcriptomic data. (H) Quantification of mitochondrial fluorescence metrics. (I,J) Cytosolic and mitochondrial aconitase activity. (K) Tissue Zn^2+^ content. (L) Tissue citrate content. (M) Volcano plot of rat salivary‐gland transcriptomic data. *n* = 15 animals per group for the in vivo study. Data are mean ± standard deviation. Statistical tests and exact *p* values are indicated in the panels.

**TABLE 2:** Body weight, water intake, salivary secretion, and salivary composition in the rat model.

Parameter	SS model rats (*n* = 15)	Control rats (*n* = 15)	*p* value
Body weight at 8 weeks (g)	200.0 ± 3.58	206.6 ± 2.87	0.072
Body weight at 11 weeks (g)	248.22 ± 3.52	250.8 ± 3.50	0.380
24 h water intake (mL)	37.8 ± 1.21	27.1 ± 0.45	< 0.0001
Salivary secretion at 10 min (g)	0.20 ± 0.012	0.56 ± 0.031	< 0.0001
Salivary secretion at 1 h (g)	1.2 ± 0.06	3.6 ± 0.18	< 0.0001
Salivary calcium (mg dL^−1^)	7.0 ± 0.15	7.3 ± 0.20	0.071
Salivary phosphorus (mg dL^−1^)	4.1 ± 0.27	4.2 ± 0.23	0.442
Salivary Zn^2+^ (µg dL^−1^)	10.0 ± 0.47	12.1 ± 0.50	0.0002
Salivary citrate (µmol dL^−1^)	2.9 ± 0.22	3.7 ± 0.27	0.001

Data are mean ± standard deviation. Salivary secretion was measured after pilocarpine stimulation.

Abbreviation: SS, Sjögren's syndrome.

MT2 and ACO2 staining increased in both salivary glands (Figure 5C,D). The same tissues showed higher reactive oxygen species (Figure 5E), altered mitochondrial morphology (Figure 5F,H), and greater cytosolic and mitochondrial aconitase activity (Figure 5I,J). Tissue Zn^2+^ and citrate were lower (Figure 5K,L), and transcriptomic profiling showed broad changes in metal‐ion and metabolic pathways (Figure 5G,M). Comparable increases in MT1/2 and ACO2 were not detected in the heart, lung, kidney, or spleen (Figure S8). The distribution of these changes was therefore concentrated in the salivary glands in this model.

### 2.5 Zinc Repletion Correlates with Higher Citrate and Improved Mineralization in SS‐Derived Epithelial Cells

The isolation and analysis workflow for primary salivary gland epithelial cells is outlined in Figure 6A. Primary salivary gland epithelial cells (SGCs) from control and SS rats displayed epithelial morphology (Figure 6B) and expressed CK19 and pan‐cytokeratin (Figure 6D,E), with no major difference in apoptosis (Figure 6C). Compared with control cells, SS‐derived SGCs showed greater mitochondrial activation (Figure 6F), lower labile Zn^2+^ (Figure 6G), lower intracellular citrate (Figure 6H), and higher ACO2 activity (Figure 6I). ACO2, MT1, MT2, and ZnT1 expression increased, whereas ZIP6 and ZIP10 expression decreased at both the mRNA and protein levels (Figure 6J,K).

**FIGURE 6:**
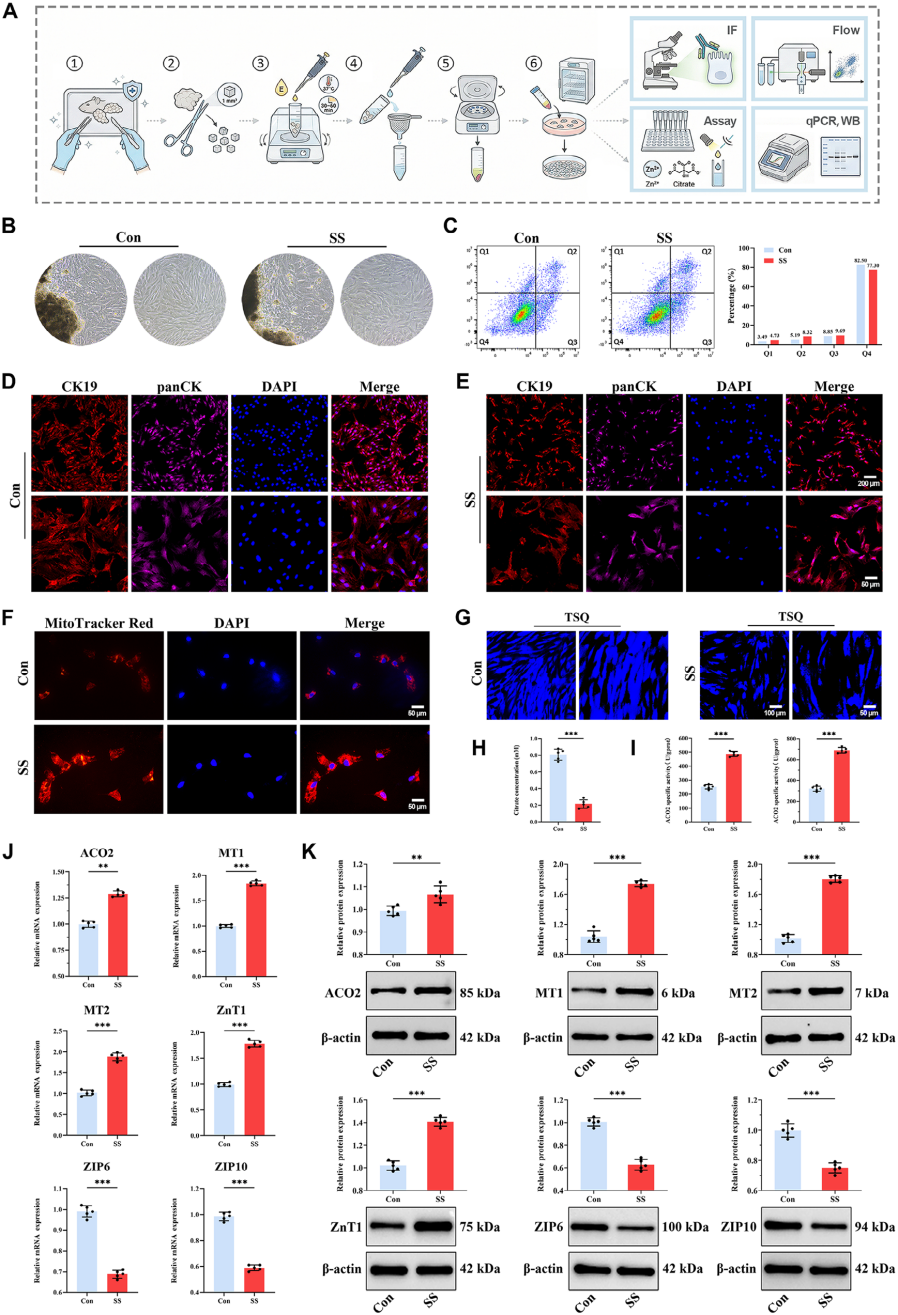
Primary salivary gland epithelial cells reproduce the zinc‐citrate metabolic phenotype. (A) Isolation and analysis workflow. (B) Bright‐field morphology. (C) Flow‐cytometric apoptosis analysis. (D, E) CK19 and pan‐cytokeratin immunofluorescence in control‐ and SS‐derived cells. (F) MitoTracker imaging. (G) TSQ imaging of labile Zn^2+^. (H) Intracellular citrate. (I) Cytosolic and mitochondrial ACO2 activities. (J) Relative mRNA expression of *ACO2*, *MT1*, *MT2*, *ZnT1*, *ZIP6*, and *ZIP10*. (K) Representative immunoblots and quantification. *n* = 5 independent cell culture runs, with three valid fields or measurements obtained per run for different panels. Data are mean ± standard deviation. Statistical tests and exact *p* values are indicated in the panels.

Exposure of SS‐derived SGCs to 50 µmol L^−1^ ZnSO_4_ for 48 h increased intracellular labile Zn^2+^, lowered ACO2 activity, and raised citrate toward control levels (Figure 7A–D). The expression levels of MT1/2, ACO2, and the zinc transporters also moved toward the control profile (Figure 7E,F). In the 7‐day mineralization assay, supernatants from ZnSO_4_‐treated SS‐derived SGCs produced more dentin‐surface mineralization and tubule occlusion (Figure 7G–J). This intervention links zinc availability with citrate preservation and with a mineralization‐relevant secretory phenotype. ZnSO_4_ affects multiple zinc‐dependent processes, so this intervention does not establish pathway specificity.

**FIGURE 7:**
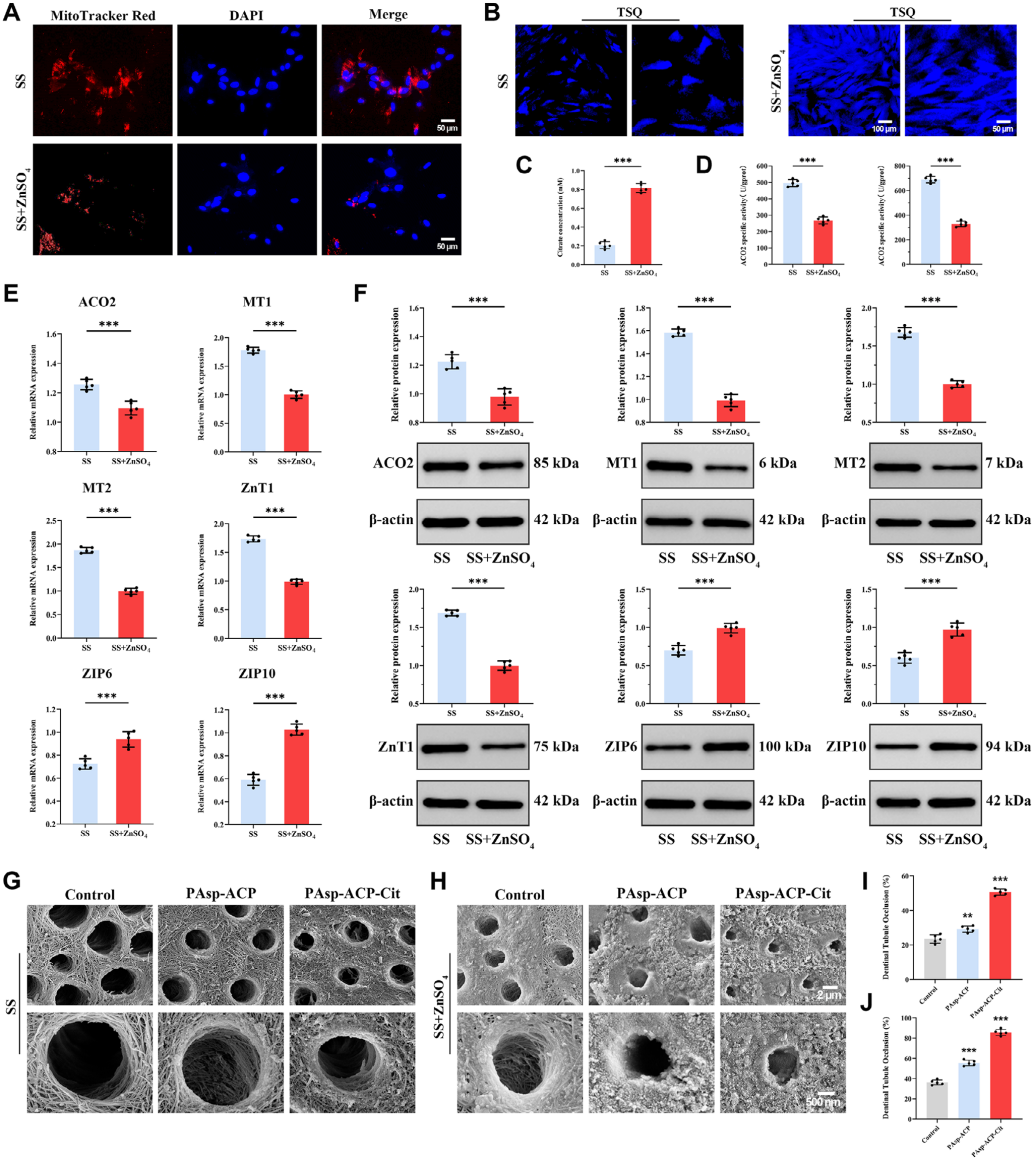
Zinc supplementation restores citrate homeostasis in SS‐derived salivary gland epithelial cells. (A) MitoTracker imaging after treatment with 50 µmol L^−1^ ZnSO_4_ for 48 h. (B) TSQ imaging of labile Zn^2+^. (C) Intracellular citrate. (D) Cytosolic and mitochondrial ACO2 activities. (E) Relative mRNA expression of *ACO2*, *MT1*, *MT2*, *ZnT1*, *ZIP6*, and *ZIP10*. (F) Representative immunoblots and quantification. (G,H) SEM of dentin surfaces. (I, J) Quantitative analysis of dentinal tubule occlusion calculated from SEM images. *n* = 5 independent cell culture runs, with three valid fields or measurements obtained per run for different panels. Data are mean ± standard deviation. Statistical tests and exact *p* values are indicated in the panels.

## 3 Discussion

Citrate deficiency was associated with impaired dentin remineralization even though the major mineral ions were preserved. Early‐stage SS provided a clinical setting in which salivary flow was also relatively maintained. Within this cohort, lower salivary citrate accompanied greater caries burden. Reproducing the disease‐associated citrate concentration in controlled mineralization systems reduced intrafibrillar mineral deposition, hydroxyapatite organization, and mechanical recovery. Human tissue, rat, and primary‐cell experiments then showed a recurring pattern of MT1/2 induction, reduced labile Zn^2+^, increased ACO2 activity, and citrate depletion. The combined observations connect altered glandular citrate metabolism with less efficient extracellular dentin mineralization.

The results distinguish mineral availability from the ability to use that mineral during repair. Salivary flow is important for clearance, buffering, and mineral delivery, yet flow rate and bulk calcium‐phosphate concentrations do not by themselves describe the remineralizing capacity of saliva. In the human cohort, citrate was markedly reduced while salivary flow and calcium, phosphate, magnesium, and bicarbonate were preserved. Citrate also ranked highest in the exploratory DMFT prediction model for the SS cohort. One interpretation is that an adequate mineral supply may be less effective when molecules involved in precursor stabilization and organization are deficient. The cross‐sectional design prevents temporal inference because DMFT reflects cumulative disease and treatment history. Lesion‐level longitudinal studies are needed to determine whether salivary citrate predicts subsequent lesion activity.

The mineralization experiments provide a direct explanation for this separation between supply and utilization. Citrate interacts with collagen, ACP, and hydroxyapatite surfaces [35, 36, 37] and can influence nucleation [38], precursor stability [39], crystal growth [40], and intrafibrillar transport [41, 42]. In reconstituted collagen, the disease‐associated citrate concentration produced sparse and poorly organized mineral, whereas the HC‐associated concentration increased intrafibrillar filling and nanoscale mechanical recovery. The same concentration difference altered crystal order, grain size, tubule occlusion, surface roughness, and modulus in demineralized dentin. Because calcium and phosphate were matched across conditions, the observed differences are consistent with a role for citrate in organizing available mineral precursors into dentin. It has been reported that citrate may also adsorb to collagen, increase fibrillar electronegativity, and recruit mineral precursors [40].

The glandular experiments offer a possible metabolic source for the extracellular citrate deficit. MT1/2 induction, zinc‐transporter remodeling, reduced labile Zn^2+^, increased ACO2 activity, and citrate depletion were observed in human tissue, the rat model, and primary SGCs. Zinc ions can inhibit mitochondrial aconitase in zinc‐accumulating cells, while metallothioneins and ZIP/ZnT transporters regulate the size and distribution of the labile Zn^2+^ pool [34, 43]. Treatment with ZnSO_4_ increased intracellular Zn^2+^, lowered ACO2 activity, restored citrate, and enhanced the mineralization activity of cell supernatants. This experiment demonstrates functional links among zinc availability, citrate metabolism, and extracellular mineralization. However, the exact sequence involving MT1/2, individual zinc transporters, and ACO2 cannot be determined from this experiment alone. Direct genetic perturbation and measurement of citrate export will be needed to resolve that sequence.

The proposed citrate‐deficient phenotype may not be limited to SS. Other inflammatory, metabolic, age‐related, or glandular conditions could alter citrate or zinc handling without causing profound hyposalivation. In those settings, mineral ions may be present while their organization into dentin is impaired. This possibility offers a testable explanation for variation in lesion behavior that is not fully captured by plaque exposure, flow rate, or bulk mineral concentrations. Future studies should assess citrate together with pH, buffering capacity, microbial acidogenicity, fluoride exposure, diet, and lesion‐level mineral dynamics. Independent disease cohorts will be necessary to determine whether citrate deficiency is a broader endotype or a feature of selected clinical contexts.

Two translational questions follow from these observations. Salivary citrate could have value as a marker of impaired endogenous remineralization, and local correction of a citrate‐deficient environment could complement established calcium‐, phosphate‐, and fluoride‐based approaches. The distinction between mineral supply and utilization may also inform caries risk assessment and biomimetic material design. Recent work spans local biofilm control, remineralization, and gas‐based therapies [44, 45, 46], providing context for future citrate‐based strategies. The current data do not justify systemic zinc supplementation or direct clinical citrate treatment. Citrate chelates calcium, and acidic citric‐acid formulations can demineralize dentin or alter bonding interfaces [36, 41, 42]. The beneficial response observed in this study depended on concentration and occurred in buffered mineralization systems. Neutral pH locally‐retained formulations, pharmacokinetic studies, and dose‐response experiments are required before clinical testing. A systemic zinc strategy would also require evaluation of toxicity, copper homeostasis, and patient‐specific zinc status.

The study has several limitations. The clinical cohort was small, exclusively female, restricted to early‐stage SS, and cross‐sectional, so independent longitudinal validation is needed. The machine‐learning analysis was exploratory and is susceptible to overfitting. Oral microbiome composition, dietary acid exposure, fluoride use, and lesion activity were not included as interacting determinants. Daily oral‐hygiene behavior and pre‐enrollment caries history were not reported as matching variables and may contribute residual confounding. The rat model developed hyposalivation and did not fully reproduce the preserved‐flow human phenotype, although the glandular zinc‐citrate abnormality was reproduced. Zinc repletion was tested without target‐specific perturbation of MT1/2, ACO2, or individual zinc transporters, and the route of citrate export from SGCs has not been resolved. In vivo evidence that restoring citrate changes lesion initiation or progression is also lacking. Prospective clinical studies and target‐specific experiments are therefore needed before the proposed mechanism can be generalized.

## 4 Conclusion

Salivary citrate deficiency impaired collagen and dentin remineralization despite preservation of the major mineral ions. Across human salivary‐gland tissue, the rat model, and primary epithelial cells, citrate depletion was associated with MT1/2 induction, lower labile Zn^2+^, and higher ACO2 activity. Zinc repletion raised intracellular citrate and improved the mineralization activity of salivary gland epithelial‐cell supernatants. These results describe a candidate remineralization failure state that differs from simple mineral shortage or severe hyposalivation. Its generalizability and therapeutic relevance now require longitudinal clinical validation, target‐specific perturbation, and in vivo caries models.

## 5 Experimental Section

### 5.1 Sex as a Biological Variable

The human cohort was restricted to women because primary SS is strongly female‐predominant. This restriction also reduced sex‐related heterogeneity in the exploratory mechanistic analyses. Female Sprague‐Dawley rats were used to align the animal experiments with the clinical cohort. Generalization to male participants or animals requires separate validation.

### 5.2 Study Participants

This cross‐sectional study enrolled 35 patients with early‐stage SS who attended The Affiliated Stomatological Hospital of Nanjing Medical University between January 2023 and December 2024 and 35 age‐ and sex‐matched healthy volunteers. SS was classified according to the 2016 American College of Rheumatology/European League Against Rheumatism criteria [47]. Inclusion criteria were diagnosis within 3 years before enrollment, female sex, age 30–35 years, no immunosuppressants, glucocorticoids, or medications affecting salivary secretion during the preceding 3 months, and no topical fluoride mouthwash or remineralizing material during the preceding month. Exclusion criteria were another autoimmune disease, severe oral infection, periodontitis, oral tumors, smoking or alcohol abuse, and severe hepatic or renal dysfunction. Age and sex were the matching variables; both cohorts comprised women aged 30–35 years.

### 5.3 Saliva Collection, Biochemical Analysis, and Caries Assessment

Unstimulated saliva was collected first. Stimulated saliva was then collected during chewing of a sterile paraffin block for 10 min. Samples were centrifuged at 3000 rpm for 10 min at 4°C; supernatants were passed through a 0.22 µm filter and stored at −80°C. Citrate was measured using a colorimetric assay (MAK057, MilliporeSigma), Zn^2+^ by inductively coupled plasma mass spectrometry (Agilent 7900), and calcium and phosphate using commercial assays (ab102505, Abcam; MAK308, MilliporeSigma). Salivary flow was determined gravimetrically and normalized to collection time. Two calibrated dentists, blinded to biochemical results, recorded DMFT, DT, MT, and FT according to World Health Organization criteria [48, 49]. Disagreements were resolved by consensus. Minor salivary‐gland tissue was obtained by lower‐lip biopsy, fixed in 4% paraformaldehyde, and processed for histology and immunostaining.

### 5.4 Exploratory Machine‐Learning Analysis

Random‐forest and XGBoost regression models were used as exploratory analyses to examine the relative contribution of measured salivary variables to DMFT. Model performance was summarized using mean squared error, root mean squared error, mean absolute error, and *R* ^2^. Shapley additive explanations (SHAP) values and model‐specific feature importance were used for interpretation. Because of the small cohort size, these analyses were considered hypothesis‐generating and were not used for confirmatory inference. Both XGBoost models used 70 participants (35 SS and 35 HC), with five‐fold cross‐validation and GridSearchCV tuning. Out‐of‐fold predictions yielded R^2^/RMSE values of 0.795/1.234 for SS and 0.885/0.650 for HC. Tuning and evaluation used the same folds without an independent test set, so performance may be optimistic; model results and SHAP rankings remain exploratory.

### 5.5 Collagen and Dentin Remineralization Models

Recombinant type‐I collagen and demineralized dentin models were established as described previously [50, 51]. Type I collagen lyophilizate (C8062, MilliporeSigma) was dissolved in 0.01 mol L^−1^ acetic acid, filtered, neutralized with phosphate‐buffered saline, and allowed to self‐assemble. Collagen suspensions were deposited on nickel grids. Sound human third molars were sectioned at low speed, ground, and polished to obtain dentin disks 1 mm thick and 5 mm in diameter. Collagen grids and dentin disks were preincubated for 48 h at 37°C in artificial saliva containing the indicated citrate concentrations and then transferred to PAsp‐ACP mineralization solutions containing matched citrate concentrations for 7 d at 37°C. The medium and PAsp‐ACP preparation followed the cited protocols [50, 51]. Citrate was varied while major mineral‐ion conditions were matched. The primary comparisons used 0.36 and 1.78 mmol L^−1^ citrate, corresponding to the mean concentrations measured in the SS and HC cohorts, respectively; intermediate concentrations were analyzed in parallel. Mineralization was evaluated by X‐ray diffraction, Fourier‐transform infrared spectroscopy, TEM, SEM, AFM, and 3D stochastic optical reconstruction microscopy. AFM roughness and Derjaguin‐Muller‐Toporov modulus were used as structural and mechanical readouts.

### 5.6 Human Salivary Gland Transcriptomic Analysis

The public salivary gland dataset GSE154926 was downloaded from the Gene Expression Omnibus and analyzed in R version 4.2.2. Differential expression was assessed using limma. Gene Ontology and Kyoto Encyclopedia of Genes and Genomes enrichment analyses were performed using clusterProfiler, and protein‐interaction networks were obtained from STRING and visualized in Cytoscape version 3.9.1.

### 5.7 Immunization‐Induced Rat Model

Female specific‐pathogen‐free Sprague‐Dawley rats aged 6–8 weeks (180–220 g) were assigned to an SS model group or control group (*n* = 15 per group). On days 0 and 7, model animals received subcutaneous injections of fresh submandibular‐gland protein emulsified with Freund's complete adjuvant. On day 14, a booster containing submandibular‐gland protein emulsified with Freund's incomplete adjuvant was administered. Controls received equal‐volume saline injections at the same sites. Model establishment was evaluated by water intake, pilocarpine‐stimulated salivary secretion, and salivary‐gland histopathology [52, 53]. After saliva collection, parotid and submandibular glands were harvested for histology, immunostaining, reactive oxygen species and mitochondrial imaging, aconitase activity, and citrate and Zn^2+^ measurements.

### 5.8 Primary Salivary Gland Epithelial Cell Isolation and Zinc Intervention

Fresh salivary‐gland tissue from control and SS rats was minced into approximately 1 mm^3^ fragments and digested with collagenase I (#17100‐017, Gibco) for 30–60 min at 37°C with gentle agitation [54]. Cell suspensions were filtered, centrifuged, and cultured in DMEM/F12 (#11330‐032, Gibco) supplemented with 10% fetal bovine serum and 1% penicillin‐streptomycin at 37°C in 5% CO_2_. Passages 3–5 were used. For rescue experiments, SS‐derived SGCs were treated with 50 µmol L^−1^ ZnSO_4_ for 48 h. Labile intracellular Zn^2+^ was evaluated with the TSQ fluorescent probe (HY‐119287, MedChemExpress), citrate with the colorimetric assay described above, and *ACO2*, *MT1*, *MT2*, *ZIP6*, *ZIP10*, and *ZnT1* by quantitative reverse‐transcription polymerase chain reaction and immunoblotting.

### 5.9 Tissue and Cellular Metabolic Assays

Cytosolic and mitochondrial aconitase activities, citrate, Zn^2+^, reactive oxygen species, and mitochondrial morphology were measured in gland tissue or SGCs using the assays indicated in the corresponding figure legends. Quantitative imaging was performed using identical acquisition settings within each experiment and analyzed in ImageJ.

### 5.10 Quantitative PCR, Immunoblotting, and Immunostaining

Total RNA was extracted with FreeZol reagent (R711, Vazyme), reverse‐transcribed using HiScript III RT SuperMix (R323‐01, Vazyme), and analyzed using SYBR Green qPCR Master Mix (Q711, Vazyme). GAPDH was the internal reference, and relative expression was calculated using the 2^‐ΔΔCt method. The qPCR experiments in Figures 6 and 7 used rat‐derived cells; primer information was verified, with corrected sequences provided in Table S1. Total protein was extracted in RIPA buffer, quantified by bicinchoninic‐acid assay, separated by SDS‐PAGE, and transferred to polyvinylidene fluoride membranes. Membranes were incubated with antibodies against MT1/2, ACO2, zinc transporters, and loading controls, followed by horseradish‐peroxidase‐conjugated secondary antibodies and chemiluminescent detection. For immunohistochemistry, paraffin sections were deparaffinized, subjected to antigen retrieval, incubated with primary and secondary antibodies, developed with 3,3′‐diaminobenzidine, and counterstained with hematoxylin. For immunofluorescence, frozen sections or cell coverslips were fixed, permeabilized, blocked, stained with the indicated antibodies and DAPI, and imaged by confocal microscopy (LSM 880, Zeiss). Antibody details are provided in Table S2.

### 5.11 Statistical Analysis

Data are presented as mean ± standard deviation unless stated otherwise. Two‐group comparisons used an unpaired two‐tailed Student's t‐test. Comparisons among more than two groups used one‐way analysis of variance followed by Tukey's multiple‐comparison test. Spearman rank correlation was used for salivary variables and caries indices. Statistical significance was defined as p < 0.05. Sample‐size calculations were performed in G*Power version 3.1 to target at least 80% power.

### 5.12 Study Approval

The human study was approved by the Ethics Committee of The Affiliated Stomatological Hospital of Nanjing Medical University (PJ‐2025‐104‐001), and all participants provided written informed consent. Animal experiments were approved by the Animal Ethics Committee of Nanjing Medical University (IACUC‐2511051) and were conducted in accordance with institutional requirements and the principles of replacement, reduction, and refinement.

## Author Contributions

**Ran You**: investigation, formal analysis, data curation, visualization, writing – original draft, and writing – review and editing. **Wenhui Wang**: investigation, validation, data curation, and writing – original draft. **Franklin R Tay**: writing – review and editing. **Haotian Dong**: investigation and visualization. **Mutong Wei**: clinical investigation, resources, and formal analysis. **Ruoyun Wu**: investigation and data curation. **Xin Song**: resources and formal analysis. **Haifeng Xie and Chen Chen**: conceptualization, supervision, project administration, funding acquisition, and writing – review and editing. All authors reviewed and approved the final manuscript.

## Funding

This work was supported by the Projects for Public Health Research in Jiangsu Province (BJ24025 and BJ25023) and the Priority Academic Program Development of Oral Medicine in Jiangsu Higher Education Institutions (JX10531804YS052507).

## Conflicts of Interest

The authors declare no conflicts of interest.

## Supporting information




**Supporting File**: advs78578‐sup‐0001‐SuppMat.docx.

## Data Availability

The public human salivary‐gland transcriptomic dataset analyzed in this study is available in the Gene Expression Omnibus under accession GSE154926. De‐identified clinical and experimental data generated in this study are available from the corresponding authors upon reasonable request and subject to applicable ethics and data‐use requirements.
