# Supplementary material for: Salivary Citrate Deficiency Defines an Endogenous Dentin Remineralization Failure State Through Glandular MT1/2‐Zn2+‐ACO2 Dysregulation
Source: Adv Sci (Weinh). 2026 Oct 29:e78578. Online ahead of print. doi: 10.1002/advs.78578 (PMC13720262; doi:10.1002/advs.78578)
Supplement: Supplementary file 1 [file ADVS-9999-e78578-s001.docx]

Intermediate citrate concentrations (0.80 and 1.20 mmol L^-1^) were evaluated to characterize the dose-response relationship between citrate availability and dentin mineralization. Fourier-transform infrared spectroscopy showed a concentration-dependent recovery of phosphate-associated mineral signals. As citrate concentration increased, the intensity of peaks corresponding to hydroxyapatite phosphate vibrations (≈1030 cm^-1^, ≈560 cm^-1^, ≈600 cm^-1^) progressively increased, while the collagen amide I profile (≈1650 cm^-1^) remained stable across groups. The PAsp-ACP-Cit system consistently achieved greater phosphate signal recovery than the PAsp-ACP system and the blank control at each matched citrate concentration, consistent with a facilitative role of citrate in ordered mineral deposition.

**Supplementary Figure 1. Fourier-transform infrared spectroscopy of demineralized dentin surfaces under intermediate citrate concentrations.** FTIR spectra of dentin slice surfaces treated with Control, PAsp-ACP, or PAsp-ACP-Cit mineralization systems under 0.80 mmol L^-1^ and 1.20 mmol L^-1^ citrate conditions (intermediate concentrations between 0.36 and 1.78 mmol L^-1^, which correspond to the mean salivary citrate concentrations measured in patients with Sjögren’s syndrome and healthy controls, respectively). Spectra are presented with amide I band normalization to show relative recovery of mineral-associated phosphate signals. Cit0.8, 0.80 mmol L^-1^ citrate; Cit1.2, 1.20 mmol L^-1^ citrate; Control, mineralization blank; PAsp-ACP, polyaspartic-acid-stabilized amorphous calcium phosphate without added citrate; PAsp-ACP-Cit, corresponding citrate-containing mineralization system.

X-ray diffraction analysis confirmed a concentration-dependent effect of citrate on hydroxyapatite crystallinity. At both 0.80 and 1.20 mmol L^-1^ citrate, the PAsp-ACP-Cit group exhibited progressively sharper characteristic hydroxyapatite diffraction peaks corresponding to the (002), (211), and (300) planes. Crystallinity and average grain size at intermediate citrate concentrations fell between the values measured at the SS-associated (0.36 mmol L^-1^) and HC-associated (1.78 mmol L^-1^) concentrations, supporting a dose-dependent regulatory effect of citrate on mineral crystal organization.

**Supplementary Figure 2. X-ray diffraction patterns of demineralized dentin surfaces under intermediate citrate concentrations.** X-ray diffraction profiles of dentin slice surfaces treated with Control, PAsp-ACP, or PAsp-ACP-Cit mineralization systems under 0.80 mmol L^-1^ and 1.20 mmol L^-1^ citrate conditions (intermediate concentrations between 0.36 and 1.78 mmol L^-1^, which correspond to the mean salivary citrate concentrations measured in patients with Sjögren’s syndrome and healthy controls, respectively). Characteristic hydroxyapatite diffraction planes (002), (211), and (300) are labeled. Cit0.8, 0.80 mmol L^-1^ citrate; Cit1.2, 1.20 mmol L^-1^ citrate; Control, mineralization blank; PAsp-ACP, polyaspartic-acid-stabilized amorphous calcium phosphate without added citrate; PAsp-ACP-Cit, corresponding citrate-containing mineralization system.

Full scanning electron microscopy was used to assess the overall uniformity of surface mineralization across the entire dentin disk surface. The extent of dentinal tubule occlusion and surface mineral coverage increased proportionally with citrate concentration. At 0.36 mmol L^-1^ citrate, most dentinal tubules remained open even in the PAsp-ACP-Cit group, with sparse and discontinuous mineral deposits. As citrate concentration increased to 0.80, 1.20, and 1.78 mmol L^-1^, tubule orifices were gradually covered by mineral deposits, and surface continuity improved. At the HC-associated concentration (1.78 mmol L^-1^), most tubules were fully occluded by a continuous mineral layer. The Control and PAsp-ACP groups showed consistently lower tubule occlusion than the PAsp-ACP-Cit group at every tested concentration.

**Supplementary Figure 3. Full scanning electron microscopy overview of dentin surfaces across all tested citrate concentrations.** Low-magnification SEM images showing global surface mineral deposition and dentinal tubule occlusion in dentin slices treated with Control, PAsp-ACP, or PAsp-ACP-Cit systems under four citrate concentrations (0.36, 0.80, 1.20, and 1.78 mmol L^-1^). Cit0.36, 0.36 mmol L^-1^ citrate (mean salivary citrate concentration measured in patients with Sjögren’s syndrome); Cit0.80, 0.80 mmol L^-1^ citrate; Cit1.20, 1.20 mmol L^-1^ citrate; Cit1.78, 1.78 mmol L^-1^ citrate (mean salivary citrate concentration measured in healthy controls); Control, mineralization blank; PAsp-ACP, polyaspartic-acid-stabilized amorphous calcium phosphate without added citrate; PAsp-ACP-Cit, corresponding citrate-containing mineralization system.

Full atomic force microscopy topography mapping corroborated the concentration-dependent effect of citrate on surface roughness. In the PAsp-ACP-Cit group, surface height variation decreased progressively with increasing citrate concentration. At 0.36 mmol L^-1^ citrate, surfaces showed substantial topographic heterogeneity with maximum height differences exceeding 3 μm, whereas at 1.78 mmol L^-1^ citrate, height differences were reduced to below 1 μm, reflecting a uniformly mineralized surface. The Control and PAsp-ACP groups exhibited limited changes in surface roughness across citrate concentrations and retained prominent surface irregularities, indicating that citrate is the primary determinant of uniform surface mineralization in this system.

**Supplementary Figure 4. Full-field AFM topography maps of dentin surfaces across all tested citrate concentrations.** Height-sensor AFM topography images of dentin slice surfaces treated with Control, PAsp-ACP, or PAsp-ACP-Cit systems under four citrate concentrations (0.36, 0.80, 1.20, and 1.78 mmol L^-1^). Quantitative vertical height ranges are indicated for each panel. Cit0.36, 0.36 mmol L^-1^ citrate (mean salivary citrate concentration measured in patients with Sjögren’s syndrome); Cit0.80, 0.80 mmol L^-1^ citrate; Cit1.20, 1.20 mmol L^-1^ citrate; Cit1.78, 1.78 mmol L^-1^ citrate (mean salivary citrate concentration measured in healthy controls); Control, mineralization blank; PAsp-ACP, polyaspartic-acid-stabilized amorphous calcium phosphate without added citrate; PAsp-ACP-Cit, corresponding citrate-containing mineralization system.

Full nanomechanical mapping demonstrated a gradient increase in Derjaguin-Muller-Toporov (DMT) modulus with rising citrate concentration. For the PAsp-ACP-Cit group, average surface modulus increased from 13.8 GPa at 0.36 mmol L^-1^ citrate to approximately 18.0 GPa at 1.20 mmol L^-1^, and reached 20.8 GPa at 1.78 mmol L^-1^. The spatial homogeneity of modulus distribution also improved at higher citrate concentrations, with high-modulus regions forming continuous domains that matched the distribution of surface mineral deposits. The Control and PAsp-ACP groups showed consistently lower modulus values than the PAsp-ACP-Cit group at each corresponding citrate concentration.

**Supplementary Figure 5. Full DMT modulus mapping of dentin surfaces across all tested citrate concentrations.** Derjaguin-Muller-Toporov modulus maps and paired height-sensor topography for dentin slice surfaces treated with Control, PAsp-ACP, or PAsp-ACP-Cit systems under four citrate concentrations (0.36, 0.80, 1.20, and 1.78 mmol L^-1^). Average modulus values and vertical height ranges are labeled for each panel. Cit0.36, 0.36 mmol L^-1^ citrate (mean salivary citrate concentration measured in patients with Sjögren’s syndrome); Cit0.80, 0.80 mmol L^-1^ citrate; Cit1.20, 1.20 mmol L^-1^ citrate; Cit1.78, 1.78 mmol L^-1^ citrate (mean salivary citrate concentration measured in healthy controls); Control, mineralization blank; PAsp-ACP, polyaspartic-acid-stabilized amorphous calcium phosphate without added citrate; PAsp-ACP-Cit, corresponding citrate-containing mineralization system.

Intermediate citrate concentrations were further tested in the reconstituted type I collagen mineralization model. At 0.80 and 1.20 mmol L^-1^ citrate, the extent of intrafibrillar mineral filling showed a clear dose-dependent pattern, with values intermediate between the SS-associated and HC-associated concentrations. Transmission electron microscopy revealed progressively denser electron-dense mineral deposits within collagen fibrils as citrate concentration increased. Selected-area electron diffraction showed increasingly defined hydroxyapatite diffraction rings, and elemental mapping confirmed enhanced calcium and phosphate co-localization within fibrils. These data reinforce the direct, concentration-dependent regulatory effect of citrate on ordered intrafibrillar mineralization.

**Supplementary Figure 6. Intermediate-concentration citrate effects on intrafibrillar mineralization of type I collagen fibrils.** TEM images, selected-area electron diffraction patterns, and calcium-phosphate elemental mapping of collagen fibrils mineralized with Control, PAsp-ACP, or PAsp-ACP-Cit systems under 0.80 mmol L^-1^ and 1.20 mmol L^-1^ citrate conditions (intermediate concentrations between 0.36 and 1.78 mmol L^-1^, which correspond to the mean salivary citrate concentrations measured in patients with Sjögren’s syndrome and healthy controls, respectively). Characteristic (004) and (211) hydroxyapatite diffraction rings are labeled. Cit0.8, 0.80 mmol L^-1^ citrate; Cit1.2, 1.20 mmol L^-1^ citrate; Control, mineralization blank; PAsp-ACP, polyaspartic-acid-stabilized amorphous calcium phosphate without added citrate; PAsp-ACP-Cit, corresponding citrate-containing mineralization system.

Hematoxylin and eosin staining was performed to verify successful establishment of the immunization-induced SS rat model. Control parotid and submandibular glands exhibited well-preserved acinar architecture, orderly cell arrangement, and normal duct morphology with minimal inflammatory cell infiltration. In contrast, SS model glands showed prominent focal lymphocytic infiltration, acinar atrophy, vacuolar degeneration of acinar cells, partial disruption of glandular architecture, and periductal inflammatory accumulation. These histopathological features are consistent with the salivary gland injury characteristic of the SS model.

**Supplementary Figure 7. Histopathological validation of the immunization-induced rat SS model by hematoxylin and eosin staining.** Representative hematoxylin and eosin staining images of parotid and submandibular gland tissues from control and SS model rats. Focal inflammatory infiltration and acinar structural damage are evident in SS gland tissues. Con, control group; SS, Sjögren’s syndrome model group.

To evaluate the tissue specificity of the MT1/2-Zn^2+^-ACO2 metabolic phenotype, immunohistochemical analysis of MT1/2 and ACO2 was performed in major non-salivary organs, including heart, lung, kidney, and spleen. No significant differences in MT1/2 or ACO2 protein expression were detected between control and SS groups in myocardial tissue, pulmonary alveolar epithelium, renal tubular epithelium, or splenic tissue. Quantitative analysis of positive staining area and mean optical density yielded comparable values between groups for all examined organs. This tissue distribution pattern indicates that the upregulation of MT1/2 and ACO2 in the SS model is predominantly restricted to salivary gland tissue, rather than representing a systemic metabolic alteration.

**Supplementary Figure 8. Tissue specificity analysis of MT1/2 and ACO2 expression in major non-salivary organs of SS rats.** Immunohistochemical staining images and quantitative analysis of MT1/2 and ACO2 in (A) myocardial tissue, (B) lung tissue, (C) renal tissue, and (D) splenic tissue from control and SS model rats. No significant intergroup differences were observed for either protein in any of the examined non-salivary organs. Con, control group; SS, Sjögren’s syndrome model group; ns, not significant. Data are mean ± standard deviation; n = 15 animals per group. Unpaired two-tailed Student’s t-test was used for two-group comparisons.

**Table S1. qPCR Primer sequences.**

| Gene name | Forward Primer | Reverse primer |
| --- | --- | --- |
| GAPDH | AGGTCGGTGTGAACGGATTTG | TGTAGACCATGTAGTTGAGGTCA |
| ACO2 | AGATGGTGCTGGAGAAGGAGAA | CCGATGGTGAAGGTGATGATG |
| MT1 | ATGGATCCCAACTGCTCCTG | GCAGCAGGCAGCTGGTCT |
| MT2 | GCTGCGCACTTGGACACTC | CGGAGGTGCAGTTACATCAGAG |
| ZIP6 | TGCTGTGGTTTCGGGTGTATG | AGTTGGTGATGGTGATGCTGG |
| ZIP10 | ACCGCTACCTGGTCCTTATCC | GGTGATGGTGAAGCGGATGA |
| ZnT1 | CTGGCTCTGTTGCTGTTGCT | CAGGTGATGGTGGTGAAGCG |

**Table S2. Antibody information.**

| Antibody | Application | Company | Cat.no | Host (Dilution) |
| --- | --- | --- | --- | --- |
| MT1/2 | IHC, IF, WB | Abcam, Cambridge, UK | ab216588 | Rabbit (IHC 1:200; IF 1:200; WB 1:1000) |
| ACO2 | IHC, WB | Abcam, Cambridge, UK | ab234682 | Rabbit (IHC 1:200; WB 1:1000) |
| PanCK | IF | Abcam, Cambridge, UK | ab80826 | Rabbit (IF 1:200) |
| CK19 | IF | Abcam, Cambridge, UK | ab52625 | Rabbit (IF 1:200) |
| ZIP6 | WB | Abcam, Cambridge, UK | ab177149 | Rabbit (WB 1:1000) |
| ZIP10 | WB | Abcam, Cambridge, UK | ab223489 | Rabbit (WB 1:1000) |
| ZnT1 | WB | Abcam, Cambridge, UK | ab221716 | Rabbit (WB 1:1000) |
| β-actin | WB | ABclonal, Wuhan, China | AC026 | Rabbit (WB 1:5000) |
| HRP-conjugated goat anti-rabbit IgG | WB | ABclonal, Wuhan, China | AS014 | Goat (WB 1:5000) |
| FITC-conjugated goat anti-rabbit IgG | IF | ABclonal, Wuhan, China | AS011 | Goat (IF 1:200) |

**Full unedited blot for Figure 6**

Original unedited Western blot bands for ACO2, MT1, MT2, ZnT1, ZIP6, ZIP10, and β-actin in primary salivary gland epithelial cells isolated from control and SS rats. Molecular weight markers are indicated on the left of each blot.

**Full unedited blot for Figure 7**

Original unedited Western blot bands for ACO2, MT1, MT2, ZnT1, ZIP6, ZIP10, and β-actin in SS-derived salivary gland epithelial cells with or without 50 μmol L^-1^ ZnSO_4_ treatment for 48 h. Molecular weight markers are indicated on the left of each blot.
